# Abnormal behavior of silica doped with small amounts of aluminum

**DOI:** 10.1038/srep35556

**Published:** 2016-10-18

**Authors:** Jinling Liu, Yiguang Wang, Linan An

**Affiliations:** 1 State Key Laboratory of Traction Power, School of Mechanics and Engineering, Southwest Jiaotong University, Chengdu, Sichuan 610031, China; 2 Science and Technology on Thermostructural Composite Materials Laboratory, Northwestern Polytechnical University, Xi’an 710072, China; 3 Department of Materials Science and Engineering, Advanced Materials Processing and Analysis Center, University of Central Florida, Orlando, FL 32816, USA

## Abstract

Silica is the most abundant mineral in the crust of the Earth. It has been demonstrated that the aluminum concentration in silica plays a key role in determining many properties of silica-based components. Although the alumina-silica system has been intensely studied, the effect of very small amounts of aluminum on the structure and properties of silica remains unclear. We report results of first principles calculations showing that small amounts of aluminum could be metastable when located in the center of Si-O rings without breaking the silica network. In contrast, higher aluminum contents will result in the destruction of the Si-O bonds, leading to the formation of triclusters and a 4-, 5-, and 6-fold Al-O coordination, as observed in previous studies. Based on the silica structure obtained through geometric optimization, the properties of silica doped with small amounts of aluminum were calculated. The results can account for many ‘abnormal’ phenomena experimentally observed. The results benefit most areas such as geosciences, microelectronics, glass industry, and ceramic materials.

Silicon dioxide is the main constituent of the earth’s crust and mantle^1^. It is also a key engineering material in many modern industries and is widely used for the fabrication of microelectronic devices, glasses, and ceramic components. Therefore, the properties and behavior of metastable, amorphous and stable silicon oxides are of fundamental interest in the geosciences^1^, microelectronics^2^, ceramics industry^3^, and materials science^4^. However, their practical application is often limited by impurities that can drastically change their mechanical, electrical^5^, and viscous properties^6^,^7^. Impurities, e.g., alkali metals^8^ or hydrogen^9^, have been demonstrated to be able to significantly affect the properties of silicon oxides.

Aluminum impurities are known to significantly affect the properties of silicon oxides, such as the viscosity of silicates^7^, the corrosion resistance^10^,^11^,^12^, and the molecular diffusion of oxygen^13^,^14^,^15^. It is generally believed that aluminum atoms can disturb the silica network, thereby decreasing the viscosity of silica and enhancing the molecular diffusion of oxygen. However, recent studies indicated that doping silica with a small amount of Al could greatly reduce the molecular diffusivity of oxygen^14^,^15^. Non-oxide ceramics with such a protective Al-doped silica layer were found to exhibit a much lower oxidation rate than those with pure silica scales^13^,^14^,^15^. Furthermore, Al-doped silica showed an exceptional corrosion-resistance in water vapor^10^,^11^,^12^. It even showed a lower weight loss by water corrosion than mullite ceramics that have very high aluminum content. Besides, the experimental results indicated that the viscosity of silica increases with the aluminum content when the aluminum concentration is in the ppm level^7^. The measured silica activities in aluminum silicate melts were also very low when the silica contained only small amounts of aluminum, even lower than the silica activities of mullite with an aluminum content of 75 at%^16^. Yet all of these phenomena are still not fully understood according to the present knowledge about aluminum silicates.

In order to better understand the properties of aluminum-doped silica, accurate knowledge of their microscopic structure is required. Previous experimental investigations^17^,^18^,^19^ have focused on the analysis of the local structure around embedded aluminum atoms. This is important for understanding the chemical ordering in aluminum-doped silicates because the Al^3+^ ions need a different environment of O^2−^ ions than the Si^4+^ ions in order to maintain the local charge balance. So far, two characteristics have been identified which distinguish the local oxygen environment of Al ions from that of Si ions: First, there are a relatively large number of fivefold and sixfold coordinated Al atoms in addition to AlO_4_ units in systems with a high alumina content, such as mullite. Second, it is evident that there exists a high amount of triclusters, which are defined as structural units where an oxygen atom is surrounded by three cations (at least one of them being an aluminum atom)^17^. Despite these progresses in our understanding of the microscopic structure of silica materials, most of the previous studies focused on silicates with high aluminum contents. In contrast, the relevant atomic-scale configuration and structure evolution process for silicate with small amounts of aluminum has yet to be determined.

## Results

**Figure 1:**
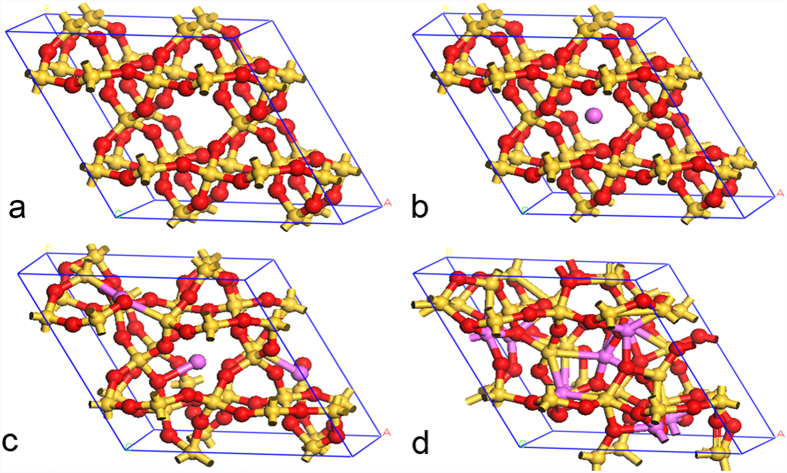
Illustration of the structural transition from quartz to metastable Al-doped silica for different doping concentrations according to the results of the *ab initio* geometric optimization. (**a**) Quartz; (**b**) Al-doped quartz (Al:Si = 1:24); (**c**) Al-doped quartz (Al:Si = 3:24); (**d**) Al-doped quartz (Al:Si = 9:24). Silicon atoms in gold, aluminum atoms in purple and oxygen atoms in red.

For the quartz structure, the metastable configurations of Al-doped quartz could be obtained after performing a geometric optimization process. As shown in Fig. 1, when the Al dopant in the silica with quartz structure, which can be described as Al/Si molar ratio, is lower than about 0.05, the whole cell expanses and the cell parameters become larger. Such a structure is metastable; no Si-O bonds are broken within the whole network. The Al ion is trapped in a cage of Si-O rings, and maintains the charge balance with the surrounding Si and O by adjusting the shape of its atomic orbitals. When the Al/Si molar ratio exceeds 0.05, the whole cell further expanses. Some Si-O rings become distorted, and some Si-O bonds are broken. The Al ion is trying to connect with O or Si to form Al-O-Si and Al-Si-O bonds. In addition, a certain amount of triclusters is formed in this case. When the Al/Si molar ratio exceeds about 0.125, the silica structure begins to collapse, resulting in a large number of broken Si-O rings. Fivefold and sixfold coordinated Al atoms are formed in addition to AlO_4_ units. At such a high aluminum content in silica, the mullite structure tends to form, which is in agreement with previous studies^14^.

**Figure 2:**
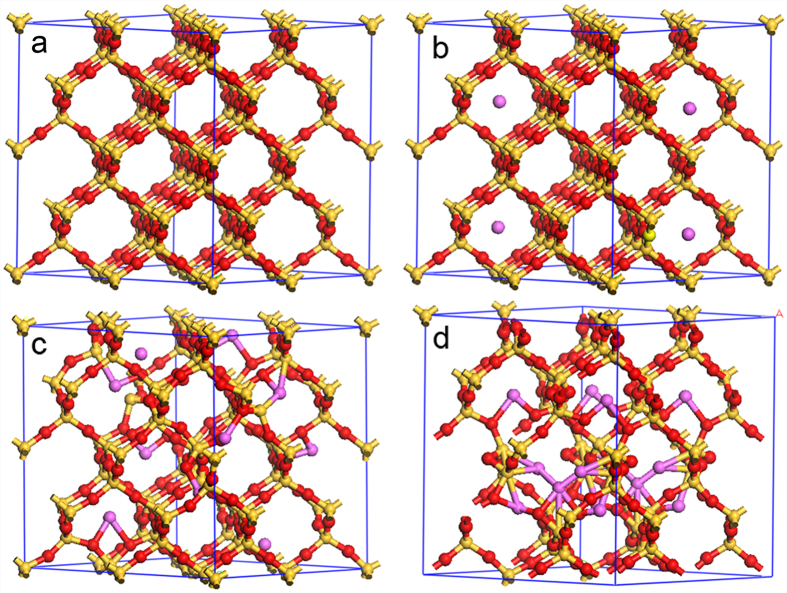
Illustration of the structural transition from cristobalite to metastable Al-doped silica for different doping concentrations according to the results of the *ab initio* geometric optimization. (**a**) Cristobalite; (**b**) Al-doped cristobalite (Al:Si = 4:64); (**c**) Al-doped cristobalite (Al:Si = 10:64); (**d**) Al-doped cristobalite (Al:Si = 14:64). Silicon atoms in gold, aluminum atoms in purple and oxygen atoms in red.

For the Al-doped cristobalite structure (Fig. 2), we observed the same kind of structural change except that the threshold Al/Si ratio for Si-O broken is slightly higher than for quartz. This may be because of the looser structure of cristobalite compared with quartz^20^. When the Al dopant in the silica with cristobalite structure, which can be described as Al/Si molar ratio, is lower than about 0.125, the structure is metastable and the aluminum sits in the Si-O cages without breaking the network. If the Al/Si molar ratio exceeds 0.125, some Si-O bonds tend to break, trying to connect with the aluminum. Triclusters are formed in addition to new bonds, i.e., Al-O-Si or Al-Si-O. A further increase of the Al/Si molar ratio will then result in the collapse of the cristobalite network and the formation of mullite.

**Figure 3:**
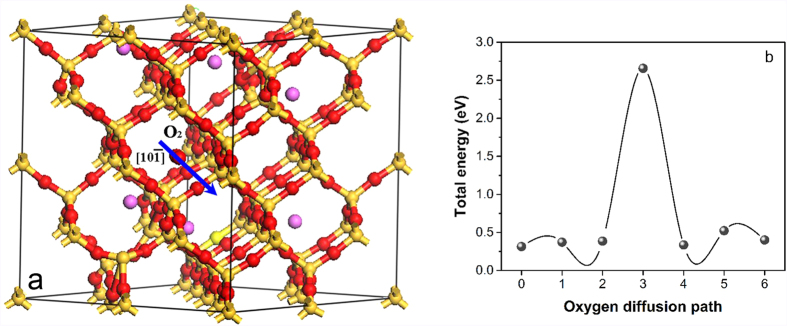
(**a**) Typical O_2_ diffusion channel in Al-doped cristobalite (silicon atoms in gold, aluminum atoms in purple and oxygen atoms in red), and (**b**) variation of the total energy along the illustrated path.

These calculations are in agreement with existing experimental results, which indicated that the silica structure expands when small amounts of aluminum are added, and that mullite tends to form in case of high aluminum concentrations^14^. This also confirms our suggestion that a small amount of aluminum within the silica structure could be metastable when embedded in the center of 6- or higher membered rings. How does such a structure affect the oxygen diffusion through the silica? Representative results for the diffusion of oxygen molecules through the ring structure of the Al-doped SiO_2_ network are shown in Fig. 3. Figure 3a shows an optimized Al-doped cristobalite structure with an Al/Si molar ratio of 6:64. An oxygen molecule is depicted to diffuse across the six-membered Si-O rings along the shaded path. These rings constitute the bottlenecks of the diffusion channel, with a 2.4 eV (Fig. 3b) energy barrier in this particular channel. Since six-membered rings are the only ring structure in cristobalite, the continuous diffusion paths are made up only from these six-membered rings. Hence, the diffusion activation energy would be 2.4 eV. This interpretation is fully consistent with the activation energies obtained through tagged ^18^O diffusion experiments^15^ and the thermal oxidation of Al-doped SiCN ceramics^13^,^14^. Compared with the results obtained for the diffusion of O_2_ in a pure silica structure (0.6–1.5 eV)^20^,^21^,^22^, the diffusion barrier is much higher in silica doped with small amounts of aluminum. The high diffusion barrier could explain the low diffusivity of oxygen molecules in case of small Al doping concentrations, as well as the abnormally high oxidation resistance of SiC^23^ and SiCN^13^,^14^ doped with small amounts of Al. According to these results, the presence of aluminum inhibits the molecular diffusion of oxygen through the network and promotes the oxidation resistance when the Al is embedded in a metastable network of 6- or higher membered Si-O rings without breaking the bonds. In contrast, high aluminum concentrations could result in a destruction of the silica network, which would promote the diffusion of oxygen. This is why many previous studies suggested that aluminum and other impurities, e.g., Na and Mg, are responsible for the deterioration of the oxidation resistance of silica^8^.

How could such Al-doped silica structures help to solve the other aforementioned dilemmas, such as the effect of aluminum on the viscosity, water vapor resistance, and activity of silica? In order to elucidate these problems, it is necessary to understand the mechanisms behind these phenomena. The recession of silica in water vapor is due to the reaction of silica with water vapor to form volatile Si(OH)_4_^24^. The Si-O bond strength could determine the extension of such a reaction. The viscosity of silica and the silica activity can also be attributed to the Si-O bond strength according to Persikov *et al*.^25^. Hence, an analysis of the strength of the Si-O bonds in Al-doped silica structures might yield the answers to the above questions. The Mulliken population analysis is the most common method to determine the electrons to be associated with the atom and the bond. The absolute magnitude of the Mulliken bond populations displays a high degree of sensitivity to the atomic basis set with which they were calculated. However, if we use the same basis set for simulating different structures, a comparison of the obtained values can provide useful information regarding the relative bond strength. According to Segall *et al*. and Winkler *et al*.^26^,^27^,^28^, the higher the Mulliken bond populations, the stronger the bonds. Thus the values obtained for the Mulliken bond populations are used to illustrate the variation of the strength of the Si-O bonds.

**Figure 4:**
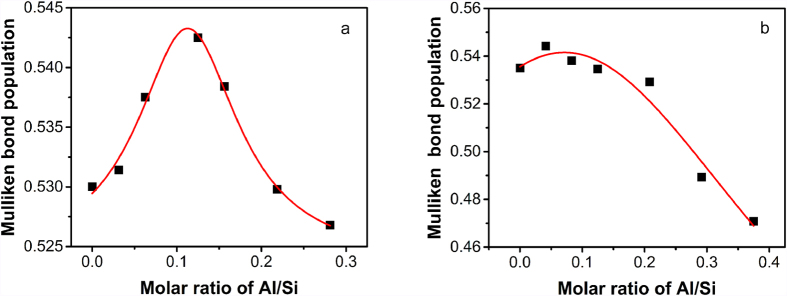
Variation of the Mulliken bond population with the Al/Si molar ratio in (**a**) the metastable Al-doped quartz and (**b**) the metastable Al-doped cristobalite.

The results obtained for quartz, shown in Fig. 4a, reveal that the presence of a small amount of aluminum increases the Mulliken bond population, indicating that the strength of the Si-O bonds is enhanced in this case. When the Al/Si molar ratio in the quartz structure is about 0.05, the Si-O bond population reaches its maximum value. When the Al/Si molar ratio exceeds 0.125, the aluminum tends to break the silica network, and the bond population becomes lower than that calculated for pure quartz. Thus the strength of the Si-O bonds deteriorates. A similar trend was also observed for the cristobalite structure (Fig. 4b). The only difference is in the percolation value of Al/Si for the Mulliken bond populations. This value is 12.5% for cristobalite, which is higher than that obtained for quartz. This is because the quartz structure is more compact than the cristobalite structure. When the Al/Si molar ratio exceeds about 0.2, the network of Si-O rings is broken up and the bond population values decrease below the values obtained for pure cristobalite. Thus the strength of the Si-O bonds will start to deteriorate. The above analysis clearly suggests that the presence of a small amount of aluminum could increase the Si-O bond strength. Therefore, it becomes clear why a small fraction of aluminum can enhance the water vapor corrosion resistance of silica. The fact that a high aluminum content could lead to the deterioration of the Si-O bond strength will help us to understand why mullite has a lower water vapor resistance despite its very high aluminum content.

## Discussion

The activity of silica in a mixture could also be determined by the strength of the Si-O bond according to alloy thermodynamics^29^. The higher the strength of Si-O bond, the lower the activity of silica. Accordingly, the strength of the Si-O bonds increases with increasing aluminum content when the aluminum concentration is sufficiently small to not break the Si-O network. The activity of silica will also decrease in this case. If the aluminum concentration exceeds a certain threshold value, the Si-O network intends to break and the strength of the Si-O bonds begins to decrease. Thus the activity of silica will increase. This is the reason why silica doped with small amounts of aluminum can exhibit a lower silica activity than mullite even though mullite contains 75 at% aluminum^16^.

According to Persikov *et al*., it is generally believed that the viscosity of silicates will increase with the Si-O bond strength^25^. Thus, the presence of a small amount of aluminum could increase the viscosity of Al-doped silica melts since it enhances the Si-O bond strength. The increase of the viscosity of silica with the aluminum content was observed in previous studies^7^, in which the aluminum concentration was in the ppm level. A higher amount of aluminum will result in the destruction of the silica network and the deterioration of the Si-O bond strength, resulting in a lower viscosity of the silicate melts. This effect of aluminum on the viscosity of silicates has been frequently observed^6^,^7^.

In summary, we calculated the effect of the aluminum concentration on the crystal structure of quartz and cristobalite and found that a small amount of aluminum could be metastable when the Al is embedded in the center of networks of 6- or higher membered Si-O rings formed by the corner-sharing SiO_4_ tetrahedra. Higher aluminum concentrations cause the silica network to break, leading to the Al forming triclusters and a 4, 5 or 6-fold coordination. The Mulliken population analysis indicated that the strength of the Si-O bonds will be enhanced by adding a small amount of aluminum to silica, and weakened by adding a large amount of aluminum. The results and conclusions presented in this work fully describe the effect of the aluminum concentration (from the ppm level to a very high content) on the properties of silica including the viscosity, the diffusion of oxygen molecules, the activity, and the water vapor corrosion resistance. The presented results are expected to benefit most areas such as microelectronics, geosciences, the glass industry, and research on ceramic materials.

## Methods

### Construction of the aluminum-doped silica model

The present silica model was set up based on the model used in our previous research on the diffusion of oxygen through Al-doped silica^14^,^15^, in which a small amount of aluminum would sit in the 6- or higher membered Si-O rings. The present choice of supercells based on the quartz and cristobalite structure was motivated by the following consideration: (i) They are typical structures, which consist of a network of only 6-, and higher membered Si-O rings with corner-sharing SiO_4_ tetrahedra; (ii) A supercell model of a vitreous system of computationally accessible size would necessarily contain many highly strained bonds, and be less representative of real vitreous SiO_2_ than a crystalline model. Accordingly, we chose the quartz supercell to contain 24 silicon atoms and 48 oxygen atoms, and the cristobalite supercell to contain 64 silicon atoms and 128 oxygen atoms. The Al-doped silica structures were then constructed by adding aluminum atoms to these supercells distributed in the channels of the 6-membered Si-O ring network.

### First-principles computer simulation

The present calculations were based on density functional theory (DFT), utilizing the CA-PZ local density approximation (LDA) method for exchange correlation, the Broyden-Fletcher-Goldfarb-Shanno (BFGS) method for the optimization algorithm, ultrasoft pseudopotentials, supercells, and plane waves^30^. The energy cutoff for the basis set was 300 eV, and the integrations over the Brillouin zone were done using the Monkhorst-Pack scheme in the relevant irreducible wedge^31^. After geometric optimization, we computed the single-point energy of the optimized structure, and analyzed the Mulliken bond populations. The distance cut-off for the bond populations was set to be 3.0 Å.

Previous studies indicated that the oxygen diffusion through the Al-doped silica was still controlled through the molecular diffusion process^15^ and that the passages for the diffusion of molecular diffusion through the silica structure were made up from 6- or higher membered Si-O rings^32^. Based on these results, we calculated the diffusion barrier for oxygen molecules through Al-doped silica. For the diffusion of oxygen molecules through the Al-filled 6-member Si-O rings, the calculated energy barrier was higher than 20 eV. This value is too high to allow for the diffusion of oxygen molecules. Therefore, the oxygen molecules are considered to diffuse through the 6-membered Si-O rings without Al inside.

## Additional Information

**How to cite this article**: Liu, J. *et al*. Abnormal behavior of silica doped with small amounts of aluminum. *Sci. Rep.* **6**, 35556; doi: 10.1038/srep35556 (2016).
